# Long noncoding RNA KCNMB2-AS1 promotes the development of esophageal cancer by modulating the miR-3194-3p/PYGL axis

**DOI:** 10.1080/21655979.2021.1973775

**Published:** 2021-09-13

**Authors:** Jiwen Xu, Xiaoyan Liu, Xueting Liu, Yunlai Zhi

**Affiliations:** a Department of Gastroenterology, Linyi Traditional Chinese Medical Hospital, Linyi, Shandong, China; b Department of Urology, The Affiliated Lianyungang Hospital of Xuzhou Medical University, The First People's Hospital of Lianyungang, Lianyungang, Jiangsu, China

**Keywords:** Esophageal cancer, KCNMB2-AS1, miR-3194-3p, PYGL

## Abstract

Esophageal cancer (ESCA), as a common cancer worldwide, is a main cause of cancer-related mortality. Comprehensive studies on molecular mechanism of ESCA have been carried out. Though numerous long noncoding RNAs (lncRNAs) was reported to participate in the occurrence and development of ESCA, the potential role of lncRNA potassium calcium-activated channel subfamily M regulatory beta subunit 2 (KCNMB2) antisense RNA 1 (KCNMB2-AS1) in ESCA remains to be discovered. This study intends to investigate the detailed function and molecular mechanism of KCNMB2-AS1 in ESCA. Gene expression was evaluated by reverse transcription quantitative polymerase chain reaction (RT-qPCR). Cell proliferation was examined by Cell Counting Kit-8 (CCK-8) assay and colony formation assay. Cell invasion and migration were measured by wound healing assay and Transwell assay. Luciferase reporter assay was adopted to validate the interaction between KCNMB2-AS1 and miR-3194-3p. Western blotting was performed to assess protein levels. We discovered that KCNMB2-AS1 was significantly upregulated in ESCA. KCNMB2-AS1 downregulation suppressed the growth, invasion, migration and stemness of ESCA cells. KCNMB2-AS1 bound with miR-3194-3p, and glycogen phosphorylase L (PYGL) was a direct target of miR-3194-3p. KCNMB2-AS1 upregulated PYGL expression by directly binding with miR-3194-3p. Additionally, PYGL overexpression abolished the inhibitory influence of KCNMB2-AS1 depletion on ESCA cell behaviors. Collectively, lncRNA KCNMB2-AS1 promotes ESCA development through targeting the miR-3194-3p/ PYGL axis, which might provide theoretical basis to explore novel biomarkers for ESCA treatment.

## Introduction

Esophageal cancer (ESCA), as one common human cancer, is also a leading cause of cancer-related mortality globally [1]. The 5-year overall survival rate of ESCA is less than 20% worldwide [2]. Much effort has been made to improve ESCA treatment [3,4]. Despite remarkable advances, the mortality of ESCA patients remains high, according to the global surveillance of trends in cancer survival during 2000–2014 [5,6]. Therefore, it is urgently required to explore the molecular mechanisms related to ESCA development and identify novel targets for ESCA treatment for better prognosis and clinical outcomes.

Long noncoding RNAs (lncRNAs) are a class of noncoding RNAs of over 200 nucleotides in length [7]. LncRNAs can act as ceRNAs by competitively binding with miRNA, thereby regulating the expression of miRNA-targeted genes [8]. LncRNAs regulate several biological processes, involving cell growth, autophagy, apoptosis, cell cycle, metastasis, and differentiation [9,10]. Increasing studies also demonstrate that the aberrant expression of lncRNAs plays a key functional role in many cancers, including ESCA [11]. Therefore, exploring the cancerogenic mechanism of lncRNAs in ESCA is significant for the etiology and optimizing treatment of ESCA. A number of lncRNAs have been validated to be dysregulated in ESCA and they can regulate the occurrence and development of ESCA, such as metastasis-associated lung adenocarcinoma transcript 1 (MALAT1) [12], actin filament associated protein 1 (AFAP1) antisense RNA 1 (AFAP1-AS1) [13], HOX transcript antisense RNA (HOTAIR) [14], taurine upregulated 1 (TUG1) [15], and maternally expressed 3 (MEG3) [16]. Through bioinformatic analysis, we identified that lncRNA KCNMB2 antisense RNA 1 (KCNMB2-AS1) is significantly upregulated in ESCA. KCNMB2-AS1 was previously reported to participate in the tumorigenesis of several human cancers. For example, KCNMB2-AS1 increases Rho associated coiled-coil containing protein kinase 1 (ROCK1) expression via binding with miR-374a-3p, therefore promoting cell proliferation, migration, and invasion and inhibiting cell apoptosis in non-small-cell lung cancer [17]. KCNMB2-AS1 is significantly overexpressed in cervical cancer, and KCNMB2-AS1 evidently facilitates tumor growth by sponging miR-130b-5p and miR-4294 and then upregulating insulin like growth factor 2 mRNA binding protein 3 (IGF2BP3) [18]. KCNMB2-AS1 enhances cell proliferation, migration, and invasion in bladder cancer through regulation of miR-374a-3p/S100 calcium binding protein A10 (S100A10) [19]. However, the role of KCNMB2-AS1 in ESCA has not yet been reported.

MicroRNAs (miRNAs) are defined as noncoding RNA molecules with lengths of approximately 17–24 nucleotides [20]. They play critical roles in diverse physiological and pathological processes by directly binding to the 3ʹ-untranslated region (3ʹ-UTR) of target genes, thereby triggering mRNA degradation or suppressing its translation [21]. Some studies have reported that dysregulated miRNAs can lead to tumorigenesis by affecting the expression of targeting genes [22–24]. For example, miR-3194-3p is significantly downregulated in hepatocellular carcinoma (HCC) tissues and cells, and miR-3194-3p inhibits the migration, invasion and epithelial-mesenchymal transition (EMT) of HCC cells via suppressing Wnt/β-catenin signaling through targeting BCL9 transcription coactivator (BCL9) [25]. MiR-3194-3p suppresses cell proliferation, migration, and invasion as well as promotes cell apoptosis in bladder cancer by targeting Aquaporin 1 (AQP1) [26]. However, the precise mechanisms of miR-3194-3p involvement in tumorigenesis of ERSC remain unclear.

The aim of this study is to investigate the role of KCNMB2-AS1 in the progression of ESCA and reveal the underlying mechanism. We hypothesized that KCNMB2-AS1 acts as oncogene in ESCA through regulating the expression of glycogen phosphorylase L (PYGL) by sponging miR-3194-3p. The results demonstrated that KCNMB2-AS1 was upregulated in ESCA and KCNMB2-AS1 promoted ESCA cell proliferation, migration, invasion as well as stemness via the miR-3194-3p/PYGL axis. This study may provide novel insights into ESCA treatment.

## Materials and methods

### Bioinformatic analysis

KCNMB2-AS1 expression in ESCA tissues versus normal tissues is predicted at Gene Expression Profiling Interactive Analysis (GEPIA) website (http://gepia2.cancer-pku.cn/) [27]. Four candidate miRNAs that can bind with KCNMB2-AS1, the top ten target mRNAs of miR-3194-3p (conditions: CLIP Data ≥ 5, Pan-Cancer ≥ 5), the expression of PYGL in ESCA tissues versus normal tissues and the binding site of KCNMB2-AS1 (or PYGL) on miR-3194-3p are demonstrated at starBase website (http://starbase.sysu.edu.cn/) [28].

### Cell culture

Human ESCA cell lines Eca109 and TE-1 and normal esophageal epithelial cell line HEsEpiC were bought from the Cell Bank of Type Culture Collection of the Chinese Academy of Sciences (Shanghai, China). All cells were incubated in Roswell Park Memorial Institute 1640 (RPMI-1640; Thermo Fisher Scientific) supplementing with 10% fetal bovine serum (FBS, Invitrogen), 100 mg/ml streptomycin and 100 U/ml penicillin at 37°C with 5% CO_2_ [29].

### Cell transfection

Short hairpin RNAs targeting KCNMB2-AS1 (KCNMB2-AS1#1/2) were transfected into ESCA cells to knockdown KCNMB2-AS1. MiR-3194-3p mimics was used to overexpress miR-3194-3p, with NC mimics as a negative control. To overexpress KCNMB2-AS1 or PYGL, the full-length sequences of KCNMB2-AS1 or PYGL was cloned into eukaryotic expression vector pcDNA3.1, with empty pcDNA3.1 as the negative control (NC). The above plasmids bought from GeneChem (Shanghai, China) were transfected into ESCA cells using Lipofectamine 3000 (Thermo Fisher Scientific) [30]. Cells transfected for 48 h were harvested for PCR analysis.

### Reverse transcription quantitative polymerase chain reaction (RT-qPCR)

Total RNA was extracted from transfected TE-1 and Eca109 cells using TRIzol reagent (Ambion) according to the manufacturer’s instructions, followed by reverse transcription to cDNAs using the GoScript Reverse Transcription System (GeneCopoeia). RT-qPCR was conducted using a SYBR Premix Ex Taq II kit (Takara, Shiga, Japan). The 2^−∆∆Ct^ method was adopted to analyze the expression of target genes [31]. U6 and Glyceraldehyde-3-phosphate Dehydrogenase (GAPDH) served as the internal references. The primers used in this study were available under requirement.

### Western blotting

Total proteins of ESCA cells was extracted by radio-immunoprecipitation assay (RIPA) lysis buffer (Beyotime) [32]. Subsequently, equal amounts of protein were transferred into polyvinylidene difluoride (PVDF) membranes after separation by 10% sodium dodecyl sulfate‐polyacrylamide gel electrophoresis (SDS-PAGE). The membranes were blocked with 5% nonfat milk and then incubated with primary antibodies against CD133 (ab222782, 1:2000, Abcam), Nanog (ab109250, 1:1000, Abcam), Oct4 (ab200834, 1:10,000, Abcam), Sox2 (ab92494, 1:1000, Abcam), ALDH1 (ab52492, 1:2000, Abcam), PYGL (ab198268, 1:1000, Abcam) and GAPDH (ab9483, 1:1000, Abcam) overnight at 4°C. GAPDH was regarded as the internal reference. Subsequently, the membranes were washed with Tris-buffered saline Tween-20 (TBST), followed by incubation with horseradish peroxidase (HRP)‐conjugated secondary antibodies for 1 h at room temperature. Finally, the enhanced chemiluminescence (ECL) kits (Abcam) were used to visualize the protein bands.

### Cell counting kit-8 (CCK-8) assay

ESCA cell growth was evaluated using Cell Counting Kit-8 (GLPBIO, Montclair, CA, USA) relative to the manufacturer’s instructions [33]. Transfected ESCA cells (1 × 10^3^) were seeded into 96-well plates and maintained in Dulbecco’s modified Eagle’s medium (DMEM) containing 10% FBS. At the time point of 24, 48, and 72 h, 10 μL of CCK‐8 solution was added to each well. Subsequently, the absorbance was detected by a microplate reader at 450 nm.

### Colony formation assay

To evaluate the proliferation of transfected ESCA cells, colony formation assay was performed as previously described [34]. In brief, cells (1 × 10^3^) were diluted and seeded into 6-well plates, followed by incubation at 37°C for 2 weeks. Then, cells were stained with 1% crystal violate (Beyotime) for 10–30 min after fixation with 4% paraformaldehyde. After 48 h, the plates were imaged, and the colony numbers in three random wells were calculated with a gel documentation system (Bio-Rad).

### Transwell assay

ESCA cell migration and invasion were evaluated using Transwell chamber (8 µm in pore diameter; Corning Glass Works, Corning, NY) [35]. Briefly, 3 × 10^5^ transfected ESCA cells were suspended in the serum-free medium and seeded on the upper compartment coated with or without Matrigel (BD Biosciences). Meanwhile, 600 μL medium containing 10% FBS was added to the lower compartment. Twenty-four h later, cells on the upper chamber were removed with cotton swabs. Cells on the lower chamber were fixed with methanol, stained with 0.1% crystal violet and photographed under an inverted microscope.

### Wound healing assay

Wound healing assay was conducted as previously described to detect the migration ability of transfected ESCA cells [36]. The indicated cells were incubated in 6-well plates. A sterile pipette tip was utilized to make wounds in each monolayer of cells when the cells were approximately 90% confluent. Next, phosphate buffer solution was utilized to wash away cell debris. At three different positions, the distance between two edges of the wound was measured. After 24 h, microscopic images were taken at the same field to evaluate the wound closure degree.

### Luciferase reporter assay

The full length of lncRNA KCNMB2-AS1 was ligated into pmirGLO luciferase reporter vectors (Promega) to construct wild-type (Wt) pmirGLO-lncRNA. PmirGLO-lncRNA mutant (Mut) was also developed in which the binding sites of miR-3194-3p were mutated. The plasmids were synthesized by Invitrogen and then cotransfected with miR-3194-3p mimics or NC mimics into TE-1 and Eca109 cells using Lipofectamine 3000 (Invitrogen). After 48 h of transfection, the Dual-Luciferase Kit (Promega) was utilized to determine the luciferase activity [37].

### RNA pulldown assay

RNA pulldown assay was carried out as previously described [38] to verify the binding capacity between KCNMB2-AS1 (or PYGL) and miR-3194-3p. After lysing, ESCA cells were treated with RNase-free DNase I (Beyotime). Afterward, the biotin-labeled wild-type miR-3194-3p (bio-miR-3194-3p Wt) or bio-NC and streptavidin-coated magnetic beads (Thermo Fisher Scientific) were incubated with cell lysate at 4°C for 3 h. Finally, RNA samples were purified by TRIzol for RT-qPCR analysis.

### Subcellular fraction assay

A PARIS™ kit (Invitrogen) was used to isolate nuclear, cytoplasmic, and total RNA [39]. Then, total RNA from the cytoplasmic and nuclear fractions was isolated with TRIzol (Invitrogen), purified and treated with DNase I, followed by reverse transcription for PCR. GAPDH served as endogenous controls for the cytoplasm, while U6 for the nucleus.

### Sphere formation assay

ESCA cells were incubated in ultra-low attachment 24-well plates (1 × 10^3^ cells/well; Corning) with DMEM/F12 medium supplemented with 20 ng·mL^−1^ epidermal growth factor (MedChemExpress, Monmouth Junction, NJ, USA), 20 ng·mL^−1^ bFGF (MedChemExpress), 1× B27 (Sigma-Aldrich, St Louis, MO, USA) and antibiotics under a humidified atmosphere with 5% CO_2_ at 37°C [40]. After 10 days, the size and number of mammospheres were evaluated and quantified under a microscope.

### Statistical analysis

Data were analyzed using SPSS21.0 (IBM Corp, Armonk, NY, USA) and presented as the mean ± standard deviation. All experiments were repeated in triplicate. Student’s *t*-test was adopted for comparisons between two groups, and one-way analysis of variance (ANOVA) followed by Tukey’s *post hoc* tests for comparisons among multiple groups. P value < 0.05 was considered statistically significant.

## Results

Our study intends to investigate the role of KCNMB2-AS1 on the progression of ESCA and reveal the possible mechanisms. We hypothesized that KCNMB2-AS1 acts as oncogene in ESCA by sponging miR-3194-3p and targeting PYGL. We carried out a series of cell function experiments in ESCA cells transfected with sh-KCNMB2-AS1 or pcDNA3.1/ KCNMB2-AS1 to evaluate the effects of KCNMB2-AS1 knockdown or overexpression on ESCA cell proliferation, migration, invasion, and stemness. The results are as follows.

### KCNMB2-AS1 knockdown suppresses cell proliferation, migration and invasion as well as stemness in ESCA

GEPIA website revealed that KCNMB2-AS1 expression was markedly elevated in ESCA tissues versus normal tissues (Figure 1(a)). As shown in RT-qPCR, KCNMB2-AS1 level was higher in ESCA cells than in normal esophageal epithelial cells (Figure 1(b)). The interfering efficiency of KCNMB2-AS1 was detected by RT-qPCR, which indicated that compared with sh-NC, sh-KCNMB2-AS1 significantly reduced KCNMB2-AS1 expression in ESCA cells (Figure 1(c)). Next, a series of loss-of-function experiments were performed to determine the effects of KCNMB2-AS1 downregulation on ESCA cell malignant behaviors. First, we discovered that downregulation of KCNMB2-AS1 markedly suppressed viability of ESCA cells compared with sh-NC (Figure 1(d)). Furthermore, sh-KCNMB2-AS1 significantly decreased colony number of ESCA cells compared with sh-NC (Figure 1(e)). The wound healing assay showed that the distance between two edges of each wound was widened after transfection of sh-KCNMB2-AS1#1/2, suggesting the inhibition of KCNMB2-AS1 silencing on ESCA cell migration (Figure 1(f)). Transwell assay demonstrated that sh-KCNMB2-AS1 repressed both the migration and invasion of ESCA cells compared with sh-NC (Figure 1(g,h)). Since tumor cell stemness is believed to be the cause tumor recurrence and metastasis, the prevention of tumor cell stemness can hinder the development of the tumor to a certain degree [41]. Then, we investigated the influence of KCNMB2-AS1 on cell stemness in ESCA. As shown by sphere formation assay, silencing of KCNMB2-AS1 reduced the number of spheroids, indicating that the sphere-forming ability of ESCA cells was attenuated (Figure 1(i)). In addition, western blotting was conducted to detect the protein levels of cancer stem cell markers (CD133, Nanog, Oct 4, Sox 2, and ALDH1), which displayed a significant decrease after KCNMB2-AS1 knockdown (Figure 1(i)). In summary, knockdown of KCNMB2-AS1 could inhibit cell proliferation, migration, and invasion as well as attenuate cell stemness in ESCA.

**Figure 1.**
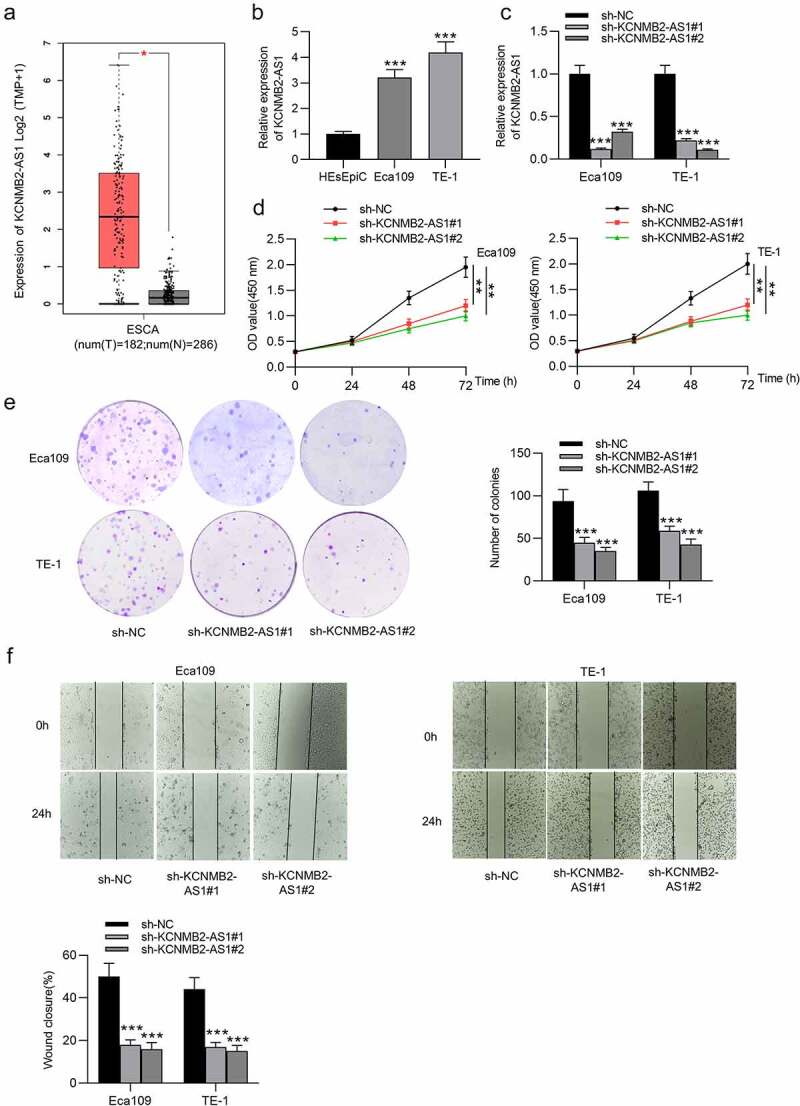
The effects of KCNMB2-AS1 knockdown on ESCA cell growth, motion, and stemness

(a) KCNMB2-AS1 expression pattern in ESCA tissues (n = 182) compared with normal tissues (n = 286) was detected from TCGA. (b) The KCNMB2-AS1 expression in ESCA cells (Eca109 and TE-1) and normal esophageal epithelial HEsEpiC cells was assesses by RT-qPCR. (c) ESCA cells were transfected with sh-KCNMB2-AS1#1/2 or sh-NC, and the interfering efficiency was confirmed by RT-qPCR. (D-E) ESCA cell proliferation after knockdown of KCNMB2-AS1 was measured by CCK-8 assay and colony formation assay. (f–h) ESCA cell migration and invasion after KCNMB2-AS1 downregulation were examined by wound healing assays and Transwell assays. (i) The number of spheroids in ESCA cells transfected with sh-KCNMB2-AS1#1/2 or sh-NC were detected by sphere formation assay. (j) The protein levels of cancer stem cell markers (CD133, Nanog, Oct 4, Sox 2, and ALDH1) in ESCA cells after downregulating KCNMB2-AS1 was evaluated by Western blotting. *p < 0.05, **p < 0.01, ***p < 0.001.

**Figure 1.**
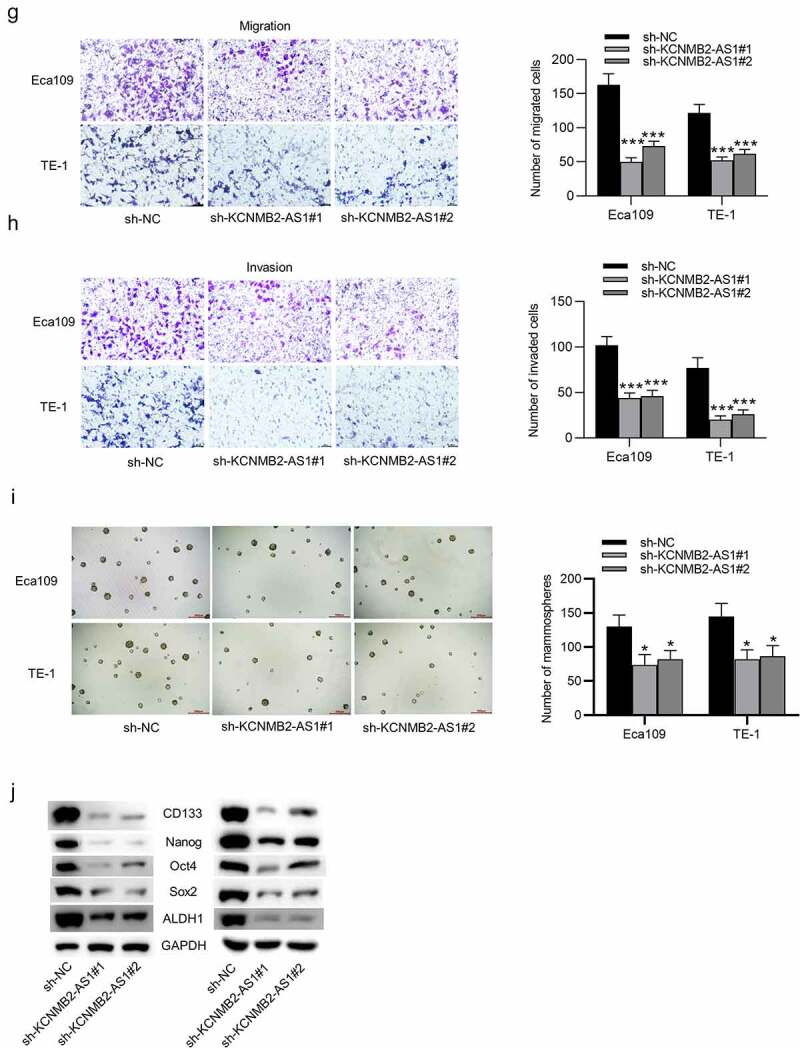
Continued

### KCNMB2-AS1 overexpression promotes cell proliferation, migration, and invasion as well as stemness in ESCA

Since the inhibitory effects of KCNMB2-AS1 knockdown were discovered on ESCA cells, then we conducted a series of gain-of-function experiments to further investigate how KCNMB2-AS1 overexpression influenced ESCA cells. The overexpression efficiency of KCNMB2-AS1 was subjected to RT-qPCR, which revealed that the expression of KCNMB2-AS1 was significantly elevated in ESCA cells after transfection with pcDNA3.1/KCNMB2-AS1 (Figure 2(a)). Then, by performing CCK-8 assay, we discovered that the viability of ESCA cells was enhanced after overexpressing KCNMB2-AS1 (Figure 2(b)). Similarly, the proliferation of ESCA cells transfected with pcDNA3.1/KCNMB2-AS1 was also promoted compared with NC group (Figure 2(c)). As shown by wound healing assay, the distance between two edges of each wound was narrowed after transfection of pcDNA3.1/KCNMB2-AS1, suggesting the overexpression of KCNMB2-AS1 facilitated ESCA cell migration (Figure 2(d)). Transwell assay demonstrated that KCNMB2-AS1 overexpression promoted both the migration and invasion of ESCA cells compared NC group (Figure 2(e,f)). In addition, the effects of KCNMB2-AS1 overexpression on ESCA cell stemness were also detected. Sphere formation assay demonstrated that overexpressing KCNMB2-AS1 increased the number of spheroids, showing that the sphere-forming ability of ESCA cells was enhanced by KCNMB2-AS1 overexpression (Figure 2(g)). Western blotting was performed to evaluate the protein levels of cancer stem cell markers (CD133, Nanog, Oct 4, Sox 2, and ALDH1), which displayed a significant increased after KCNMB2-AS1 overexpression (Figure 2(h)). Overall, KCNMB2-AS1 overexpression promotes cell proliferation, migration, and invasion as well as stemness in ESCA.

**Figure 2.**
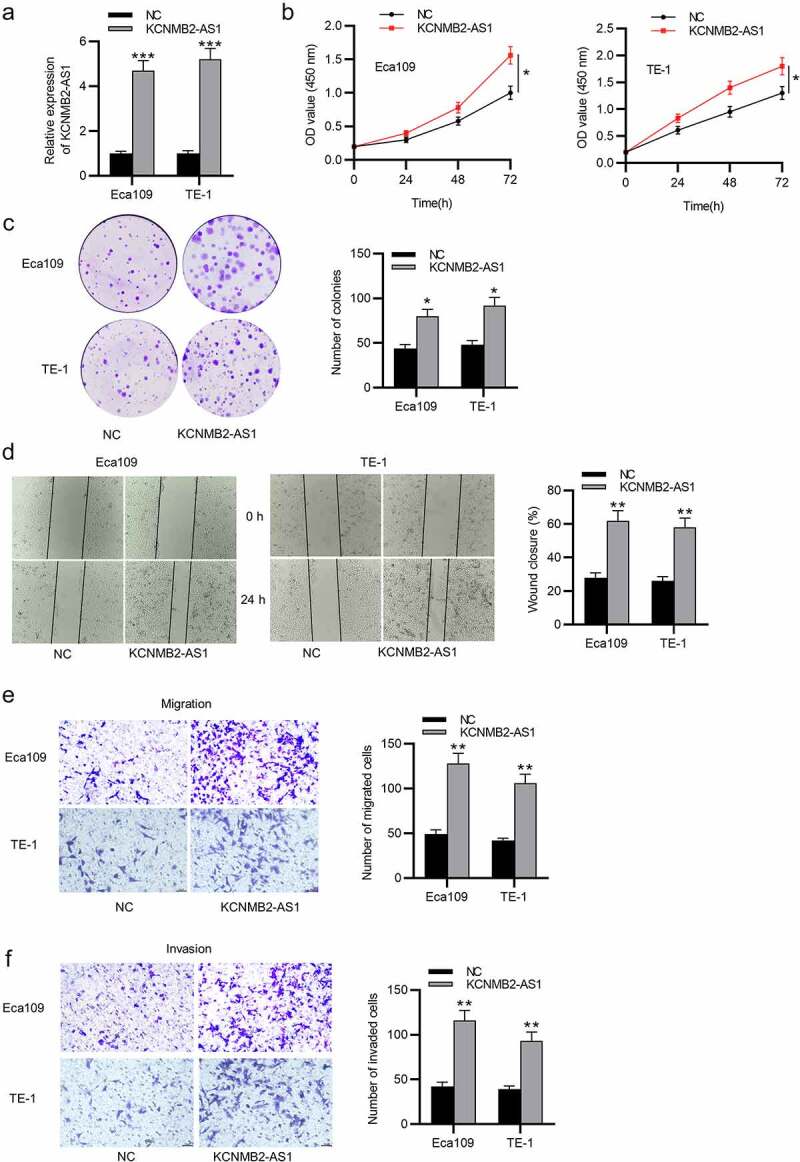
The effects of KCNMB2-AS1 overexpression on ESCA cell growth, motion, and stemness

(a) ESCA cells were transfected with pcDNA3.1/KCNMB2-AS1 or pcDNA3.1, and the overexpression efficiency was detected by RT-qPCR. (b-c) The proliferation of ESCA cells after overexpressing KCNMB2-AS1 was assessed by CCK-8 assay and colony formation assay. (d-f) ESCA cell migration and invasion after KCNMB2-AS1 overexpression were examined by wound healing assays and Transwell assays. (g) The number of spheroids in ESCA cells transfected with pcDNA3.1/KCNMB2-AS1 or pcDNA3.1 were detected by sphere formation assay. (h) The protein levels of cancer stem cell markers (CD133, Nanog, Oct 4, Sox 2, and ALDH1) in ESCA cells after overexpressing KCNMB2-AS1 was evaluated by Western blotting. *p < 0.05, **p < 0.01, ***p < 0.001.

**Figure 2.**
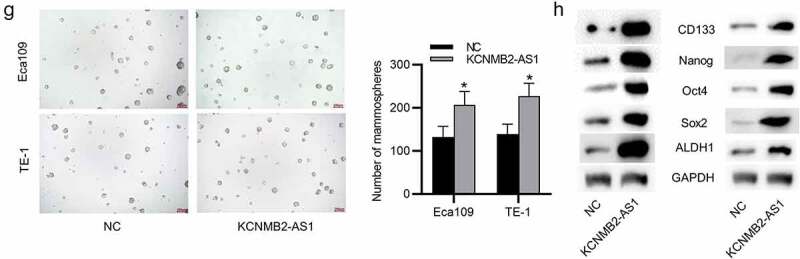
Contined

### KCNMB2-AS1 binds with miR-3194-3p

**Figure 3.**
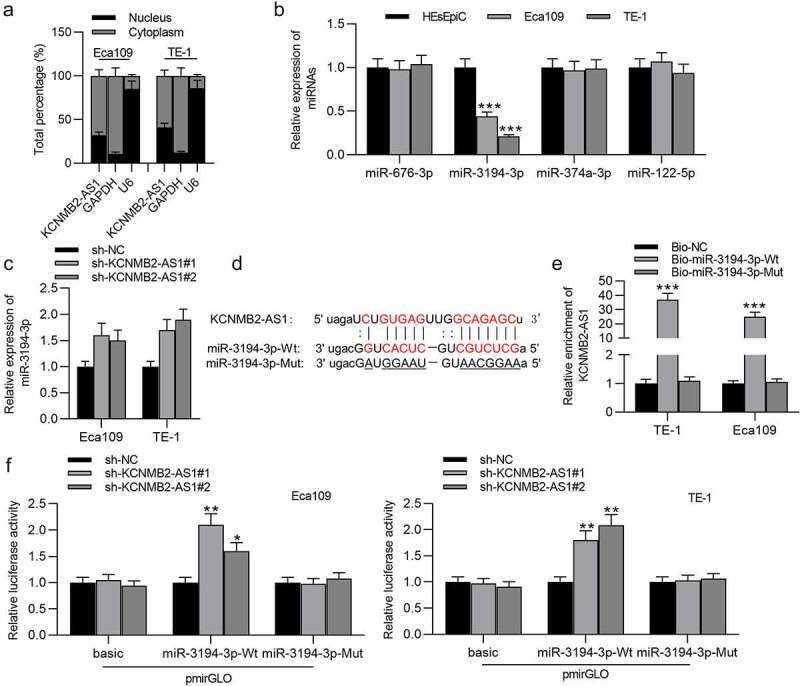
KCNMB2-AS1 binds with miR-3194-3p

To figure out the localization of KCNMB2-AS1 in ESCA cells, a subcellular fractionation assay was conducted. We discovered that KCNMB2-AS1 was mainly localized in the cytoplasm of ESCA cells (Figure 3(a)). StarBase database was utilized to search possible miRNAs that bind with KCNMB2-AS1. Four miRNAs were identified, among which only miR-3194-3p was significantly downregulated in ESCA cells (Figure 3(b)). MiR-3194-3p expression in ESCA cells transfected with sh-KCNMB2-AS1#1/2 or sh-NC were detected using RT-qPCR, which indicated that sh-KCNMB2-AS1 significantly elevated miR-3194-3p expression compared with sh-NC (Figure 3(c)). The binding site of KCNMB2-AS1 on miR-3194-3p is predicted at starBase website, and binding sequence of miR-3194-3p was mutated for the following assays (Figure 3(d)). An RNA pulldown assay was conducted. KCNMB2-AS1 expression in ESCA cells was markedly increased in bio-miR-3194-3p-Wt, while there existed no significant changes in bio-miR-3194-3p-Mut (Figure 3(e)). The binding between KCNMB2-AS1 and miR-3194-3p was further validated by a luciferase reporter assay, which revealed that the luciferase activity of miR-3194-3p-Wt plasmid was significantly elevated in both Eca109 and TE-1 cells due to KCNMB2-AS1 knockdown while no significant changes happened in the basic and miR-3194-3p Mut group (Figure 3(f)). In conclusion, KCNMB2-AS1 binds with miR-3194-3p and negatively regulates miR-3194-3p level in ESCA.

(a) The localization of KCNMB2-AS1 in ESCA cells was determined by a subcellular fractionation assay. (b) Possible miRNAs that bind with KCNMB2-AS1 were identified by starBase website. The level of the candidate miRNAs in ESCA cells compared to normal cells were detected using RT-qPCR. (c) The influence of KCNMB2-AS1 knockdown on the expression of miR-3194-3p in ESCA cells was assessed by RT-qPCR. (d) The potential binding site of KCNMB2-AS1 on miR-3194-3p is shown at starBase website. (e) The RNA pulldown assay was applied to explore whether KCNMB2-AS1 can bind with miR-3194-3p. (f) A luciferase reporter assay was conducted to further verify the relationship between KCNMB2-AS1 and miR-3194-3p. *p < 0.05, **p < 0.01, ***p < 0.001.

### PYGL is targeted by miR-3194-3p

**Figure 4.**
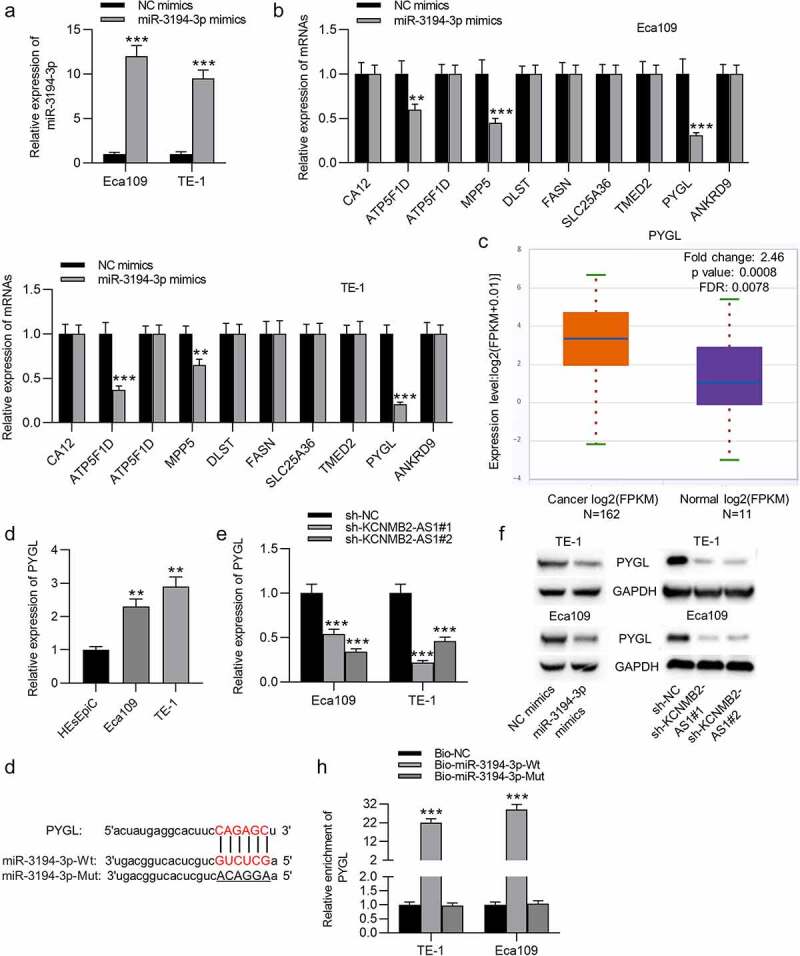
PYGL is targeted by miR-3194-3p

The overexpression efficiency of miR-3194-3p in Eca109 and TE-1 cells was verified by PCR (Figure 4a). Ten mRNAs were identified to be the underlying targets of miR-3194-3p using the starBase database. In ESCA cells transfected with miR-3194-3p mimics, PYGL expression showed the most significant downregulation compared to that of the other candidate mRNAs (Figure 4b), so PYGL was selected as the research object. Based on starBase prediction, PYGL was upregulated in ESCA tissues compared with normal tissues (Figure 4(c)). The expression of PYGL in ESCA cells was markedly higher than in normal cells (Figure 4(d)). RT-qPCR showed that KCNMB2-AS1 downregulation significantly attenuated the level of PYGL in ESCA cells (Figure 4(e)). In western blotting, the protein level of PYGL was markedly decreased in ESCA cells transfected with sh-KCNMB2-AS1 or miR-3194-3p mimics (Figure 4(f)). The possible binding site of PYGL on miR-3194-3p is shown at starBase website (Figure 4(g)). An RNA pulldown assay was performed to validate the binding between miR-3194-3p and PYGL, which revealed that PYGL expression in ESCA cells was markedly increased in bio-miR-3194-3p-Wt, with no significant change in bio-miR-3194-3p-Mut (Figure 4(h)). Overall, miR-3194-3p targets PYGL in ESCA.

(a) The overexpression efficiency of miR-3194-3p in ESCA cells was detected using RT-qPCR. (b) Ten mRNAs were identified to be targets of miR-3194-3p using the starBase database. RT-qPCR was performed to examine the expression of these ten mRNAs in ESCA cells transfected with miR-3194-3p mimics or NC mimics. (c) PYGL expression in ESCA tissues (n = 162) relative to normal tissues (n = 11) was predicted at starBase website. (d) RT-qPCR was used to assess the expression of PYGL in ESCA cells and normal esophageal epithelial cells. (e) RT-qPCR was conducted to evaluate how KCNMB2-AS1 knockdown influenced PYGL expression in ESCA cells. (f) PYGL protein levels in ESCA cells transfected with NC mimics, miR-3194-3p mimics, sh-NC, and sh-KCNMB2-AS1 #1/2 were assessed by Western blotting. (g) The possible binding site of PYGL on miR-3194-3p is shown at starBase website. (h) An RNA pulldown assay was carried out to validate the relationship between PYGL and miR-3194-3p. **p < 0.01, ***p < 0.001.

### Overexpression of PYGL reverses sh-KCNMB2-AS1 induced inhibitory effects on ESCA cell behavior

Finally, rescue experiments were conducted to explore whether KCNMB2-AS1 affects ESCA development by regulating PYGL. First, ESCA cells were transfected with pcDNA3.1/PYGL or pcDNA3.1, and the overexpression efficiency was detected by RT-qPCR and western blotting (Figure 5(a,b)). CCK-8 assays and colony formation assays demonstrated that sh-KCNMB2-AS1 attenuated the growth of ESCA cells relative to sh-NC, while cotransfection of sh-KCNMB2-AS1 and pcDNA3.1/PYGL rescued sh-KCNMB2-AS1 induced inhibitory influence on cell growth (Figure 5(c,d)). In addition, sh-KCNMB2-AS1 alleviated the migratory and invasive capacities of ESCA cells, while co-transfection of sh-KCNMB2-AS1 and pcDNA3.1/PYGL obviously reversed the inhibited invasion and migration caused by sh-KCNMB2-AS1 (Figure 5(e,g)). Furthermore, the reduced number of spheroids caused by KCNMB2-AS1 knockdown was reversed by PYGL overexpression (Figure 5(h). Western blotting demonstrated that the protein levels of cancer stem cell markers (CD133, Nanog, Oct 4, Sox 2, and ALDH1) in ESCA cells were decreased after transfection with sh-KCNMB2-AS1, but rescued after cotransfection with sh-KCNMB2-AS1 and pcDNA3.1/PYGL (Figure 5(i)). Taken together, overexpression of PYGL abolished the inhibitory influence of KCNMB2-AS1 knockdown on ESCA stemness. Therefore, we concluded that KCNMB2-AS1 facilitates ESCA development by upregulating PYGL.

**Figure 5.**
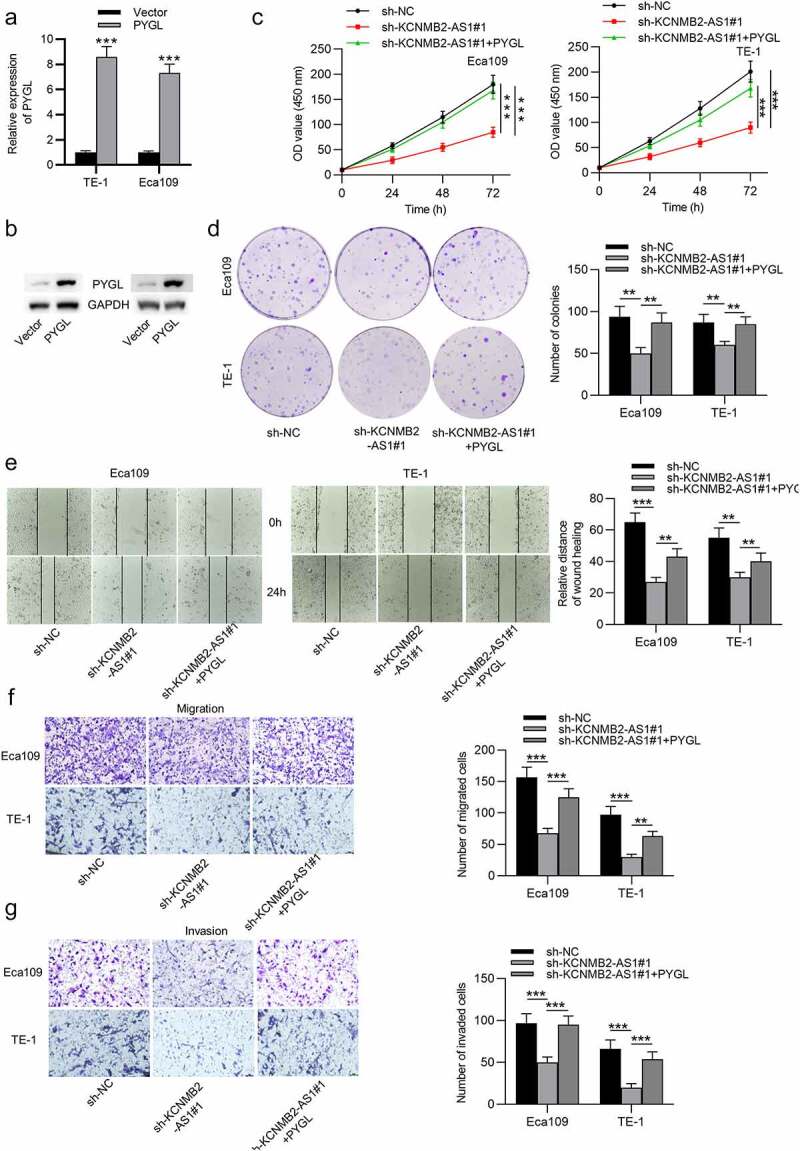
Overexpression of PYGL reverses inhibitory effects of KCNMB2-AS1 knockdown on ESCA cell behavior

(a–b) RT-qPCR and western blotting were performed to detect the overexpression efficiency of KCNMB2-AS1 in ESCA cells. (c–d) CCK-8 assay and colony formation assay were conducted to detect ESCA cell proliferation in 3 groups: sh-NC group, sh-KCNMB2-AS1 group, sh-KCNMB2-AS1+ PYGL group. (e) Wound healing assay was performed to assess cell migration in the above groups. (f–g) Transwell assays were carried out to measure cell migration and invasion in the above groups. (h) Sphere formation assay was applied to assess the sphere-formation ability of ESCA cells in the above groups. (i) Western blotting was used to evaluate the protein levels of cancer stem cell markers (CD133, Nanog, Oct 4, Sox 2, and ALDH1) in ESCA cells in the above groups. *p < 0.05, **p < 0.01, ***p < 0.001.

**Figure 5.**
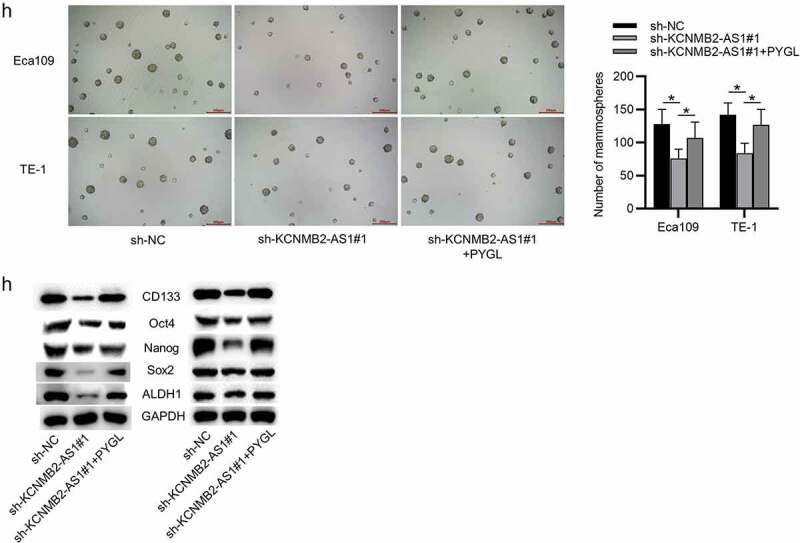
Continued

## Discussion

ESCA is one of the most aggressive cancer types globally [42], with its etiology poorly understood. Many lncRNAs have been reported to be related to various cellular processes involving proliferation, invasion, migration in human cancers [43]. In the current study, we intended to investigate whether KCNMB2-AS1 affects ESCA tumorigenesis, and the key findings indicated that KCNMB2-AS1 facilitated ESCA cell proliferation, migration, invasion, and stemness by targeting the miR-3194-3p/ PYGL axis.

The most canonical theory for the mechanism of lncRNAs is that they serve as miRNA ‘sponges’, acting as miRNA absorbers to specifically attenuate miRNA abundance [9]. Many lncRNAs are observed differentially expressed in ESCA and their molecular mechanisms have been elucidated. For example, lncRNA small nucleolar RNA host gene 7 (SNHG7) facilitates the growth and attenuates apoptosis of ESCA cells by mediating p15 and p16 levels [44]. LncRNA DEAD/H-box helicase 11 (DDX11) antisense RNA 1 (DDX11-AS1) silencing significantly inhibits the proliferation, migration and invasion of ESCA cells, and induces the level of cell apoptosis through regulating the miR-514b-3p/ring-box 1 (RBX1) axis [45]. LncRNA breast cancer antiestrogen resistance 4 (BCAR4) knockdown induces cell apoptosis and G1/S arrest, while represses cell proliferation and migration in ESCA by sponging miR-139-3p to upregulate ELAV like RNA binding protein 1 (ELAVL1) [46]. Recent studies suggested KCNMB2-AS1 as an oncogenic lncRNA in human cancers, for instance, it was reported to facilitate cell growth and motion, and repress apoptosis in nonsmall-cell lung cancer [17]. In this study, KCNMB2-AS1 was discovered significantly upregulated in ESCA. Furthermore, KCNMB2-AS1 knockdown inhibited ESCA cell growth and motion, and KCNMB2-AS1 overexpression promoted ESCA cell growth and motion, suggesting an oncogenic role of KCNMB2-AS1 in ESCA. In addition, tumor cell stemness is believed to be the cause tumor recurrence and metastasis, and the prevention of tumor cell stemness can hinder the development of the tumor to a certain degree [41]. Several lncRNAs such as FMR1 antisense RNA 1 (FMR1-AS1) [47], small nucleolar RNA host gene 12 (SNHG12) [48], HLA complex P5 (HCP5) [49], fer-1 like family member 4 (pseudogene) (FER1L4) [50] were reported to regulate ESCA cell stemness. Previously, knockdown of KCNMB2-AS1 was also reported to diminish cell stemness in bladder cancer [50]. Therefore, we also investigated whether KCNMB2-AS1 dysfunction influences ESCA cell stemness. We discovered that the sphere-formation ability of ESCA cells was attenuated and the protein levels of cancer stem cell markers (CD133, Nanog, Oct 4, Sox 2, and ALDH1) in ESCA cells were decreased after KCNMB2-AS1 downregulation, suggesting that KCNMB2-AS1 downregulation diminished ESCA cell stemness. In contrast, ESCA cell stemness was facilitated after KCNMB2-AS1 overexpression. These finding are in consistent with that in the previous study.

LncRNAs located in the cytoplasm can act as ceRNAs by competitively binding with miRNA, thereby influencing the expression of miRNA-targeted genes [51,52], and play key regulatory roles in the progression of human cancers [53]. In this study, KCNMB2-AS1 was detected mainly located in cytoplasm, implying its role as a ceRNA in ESCA. We identified that KCNMB2-AS1 bound with miR-3194-3p in ESCA. MiR-3194-3p was reported to suppress the tumorigenesis of human cancers. For example, miR-3194-3p suppresses cell invasion and growth by targeting AQP1 in breast cancer [26]. MiR-3194-3p represses the stemness and EMT of hepatocellular carcinoma cells through targeting BCL9 [25]. Herein, we discovered that miR-3194-3p level was elevated by KCNMB2-AS1 knockdown in ESCA cell lines, suggesting that KCNMB2-AS1 could affect ESCA cell behaviors by interacting with miR-3194-3p.

To further investigate the ceRNA regulatory mechanism, we examined the target genes of miR-3194-3p. Bioinformatic analysis displayed that PYGL was a candidate target of miR-3194-3p. PYGL is a gene signature derived from head and neck squamous cell carcinomas, which defines the hypoxia ‘metagene’ [54]. PYGL is upregulated in several cancers, such as seminoma, brain cancer and papillary renal cell carcinoma, as shown at Oncomine website (https://www.oncomine.org/). In the previous study, increased PYGL levels were analyzed to be linked with increased tumor size by utilizing mouse model of breast cancer, suggesting that PYGL participate in tumor progression [55]. Furthermore, PYGL depletion also decreases growth rate of cancer cells, induces cell cycle arrest and inhibits apoptosis in breast cancer, suggesting its oncogenic role [55]. Then, in the present study, PYGL was also found upregulated in ESCA. The expression and protein level of PYGL were decreased in ESCA cells after KCNMB2-AS1 downregulation or miR-3194-3p overexpression. Additionally, PYGL overexpression abolished the inhibitory influence of KCNMB2-AS1 downregulation on ESCA cell proliferation, invasion, migration and stemness. Since KCNMB2-AS1 was discovered to bind with miR-3194-3p and negatively regulate its expression, we concluded that KCNMB2-AS1 facilitated ESCA development via binding with miR-3194-3p and further upregulating PYGL expression, which is in consistent with the previous findings.

However, there were several limitations in this study. First, further investigation on the potential signaling pathways related to the detailed mechanism regarding how KCNMB2-AS1 modulates PYGL requires to be conducted in future studies. Second, whether KCNMB2-AS1 promotes tumor growth *in vivo* by targeting PYGL remains to be explored. Third, the issue that KCNMB2-AS1 might have different effects on different stages of ESCA has not been investigated in the current study. In future studies, paired tissue specimens from patients with ESCA will be collected, and the association between KCNMB2-AS1 expression and clinical characteristics (including TNM stages) in ESCA will be explored.

## Conclusion

To sum up, our findings indicated that lncRNA KCNMB2-AS1 promoted cell proliferation, migration, invasion as well as cell stemness in ESCA via targeting the miR-3194-3p/PYGL axis. This study enhanced our understanding of the pathogenesis of ESCA and demonstrated that KCNMB2-AS1 might serve as a promising diagnostic biomarker and therapeutic target for ESCA treatment.

## References

[1] Ferlay J, Steliarova-Foucher E, Lortet-Tieulent J, et al. Cancer incidence and mortality patterns in Europe: estimates for 40 countries in 2012. Eur J Cancer. 2013 Apr;49(6):1374–1403. 23485231 10.1016/j.ejca.2012.12.027

[2] Zhang SK, Guo LW, Chen Q, et al. The association between human papillomavirus 16 and esophageal cancer in Chinese population: a meta-analysis. BMC Cancer. 2015;15(1):1096. 25777422 10.1186/s12885-015-1096-1 PMC4352244

[3] Hayano K, Ohira G, Hirata A, et al. Imaging biomarkers for the treatment of esophageal cancer. World J Gastroenterol. 2019 Jun 28;25(24):3021–3029. 31293338 10.3748/wjg.v25.i24.3021 PMC6603816

[4] Watanabe M, Otake R, Kozuki R, et al. Recent progress in multidisciplinary treatment for patients with esophageal cancer. Surg Today. 2020 Jan;50(1):12–20. 31535225 10.1007/s00595-019-01878-7 PMC6952324

[5] Le Bras GF, Farooq MH, Falk GW, et al. Esophageal cancer: the latest on chemoprevention and state of the art therapies. Pharmacol Res. 2016 Nov;113(Pt A):236–244. 27565381 10.1016/j.phrs.2016.08.021 PMC5107116

[6] Smyth EC, Lagergren J, Fitzgerald RC, et al. Oesophageal cancer. Nat Rev Dis Primers. 2017 Jul;27(3):17048. 10.1038/nrdp.2017.48 PMC6168059 28748917

[7] Ulitsky I, Bartel DP. lincRNAs: genomics, evolution, and mechanisms. Cell. 2013 Jul 3;154(1):26–46. 23827673 10.1016/j.cell.2013.06.020 PMC3924787

[8] Zhang X, Wang J, Liu Z, et al. LINC00657/miR-26a-5p/CKS2 ceRNA network promotes the growth of esophageal cancer cells via the MDM2/p53/Bcl2/Bax pathway. 2020; 40(6):BSR20200525. 10.1042/BSR20200525 PMC7268253 32426838

[9] Pauli A, Rinn JL, Schier AF. Non-coding RNAs as regulators of embryogenesis. Nat Rev Genet. 2011 Feb;12(2):136–149. 21245830 10.1038/nrg2904 PMC4081495

[10] Prensner JR, Chinnaiyan AM. The emergence of lncRNAs in cancer biology. Cancer Discov. 2011 Oct;1(5):391–407. 22096659 10.1158/2159-8290.CD-11-0209 PMC3215093

[11] Kotake Y, Nakagawa T, Kitagawa K, et al. Long non-coding RNA ANRIL is required for the PRC2 recruitment to and silencing of p15(INK4B) tumor suppressor gene. Oncogene. 2011 Apr 21;30(16):1956–1962. 21151178 10.1038/onc.2010.568 PMC3230933

[12] Wang X, Li M, Wang Z, et al. Silencing of long noncoding RNA MALAT1 by miR-101 and miR-217 inhibits proliferation, migration, and invasion of esophageal squamous cell carcinoma cells. J Biol Chem. 2015 Feb 13;290(7):3925–3935. 25538231 10.1074/jbc.M114.596866 PMC4326802

[13] Luo HL, Huang MD, Guo JN, et al. AFAP1-AS1 is upregulated and promotes esophageal squamous cell carcinoma cell proliferation and inhibits cell apoptosis. Cancer Med. 2016 Oct;5(10):2879–2885. 27577754 10.1002/cam4.848 PMC5083742

[14] Ge XS, Ma HJ, Zheng XH, et al. HOTAIR, a prognostic factor in esophageal squamous cell carcinoma, inhibits WIF-1 expression and activates Wnt pathway. Cancer Sci. 2013 Dec;104(12):1675–1682. 24118380 10.1111/cas.12296 PMC7653522

[15] Xu C, Guo Y, Liu H, et al. TUG1 confers cisplatin resistance in esophageal squamous cell carcinoma by epigenetically suppressing PDCD4 expression via EZH2. Cell Biosci. 2018;8(1):61. 30519392 10.1186/s13578-018-0260-0 PMC6263046

[16] Ma J, Li TF, Han XW, et al. Downregulated MEG3 contributes to tumour progression and poor prognosis in oesophagal squamous cell carcinoma by interacting with miR-4261, downregulating DKK2 and activating the Wnt/β-catenin signalling. Artif Cells Nanomed Biotechnol. 2019 Dec;47(1):1513–1523. 30990378 10.1080/21691401.2019.1602538

[17] Yang H, Wang Z, Wang Z. Long noncoding RNA KCNMB2-AS1 increases ROCK1 expression by sponging microRNA-374a-3p to facilitate the progression of Non-Small-Cell lung cancer. Cancer Manag Res. 2020;12:12679–12695. 33335424 10.2147/CMAR.S270646 PMC7737946

[18] Zhang Y, Wang D, Wu D, et al. Long noncoding RNA KCNMB2-AS1 stabilized by N-Methyladenosine modification promotes cervical cancer growth through acting as a competing endogenous RNA. Cell Transplant. 2020;29:963689720964382. 10.1177/0963689720964382 PMC7784579 33028109

[19] Zhu J, Huang Y, Zhang Y, et al. KCNMB2-AS1 promotes bladder cancer progression through sponging miR-374a-3p to upregulate S100A10. Front Genet. 2021;12:655569. 10.3389/fgene.2021.655569 PMC8339911 34367236

[20] Saliminejad K, Khorram Khorshid HR, Soleymani Fard S, et al. An overview of microRNAs: biology, functions, therapeutics, and analysis methods. J Cell Physiol. 2019 May;234(5):5451–5465. 30471116 10.1002/jcp.27486

[21] Correia de Sousa M, Gjorgjieva M, Dolicka D, et al. Deciphering miRNAs’ action through miRNA editing. Int J Mol Sci. 2019 Dec 11;20(24):6249. 10.3390/ijms20246249 PMC6941098 31835747

[22] Cantini L, Isella C, Petti C, et al. MicroRNA-mRNA interactions underlying colorectal cancer molecular subtypes. Nat Commun. 2015 Nov;17(6):8878. 10.1038/ncomms9878 PMC4660217 27305450

[23] Chen Y, Wu Y, Yu S, et al. Deficiency of microRNA-628-5p promotes the progression of gastric cancer by upregulating PIN1. Cell Death Dis. 2020 Jul 23;11(7):559. 32703934 10.1038/s41419-020-02766-6 PMC7378826

[24] Li Y, Wang Y, Kong R, et al. Dihydroartemisinin suppresses pancreatic cancer cells via a microRNA-mRNA regulatory network. Oncotarget. 2016 Sep 20;7(38):62460–62473. 27613829 10.18632/oncotarget.11517 PMC5308739

[25] Yao B, Li Y, Wang L, et al. MicroRNA-3194-3p inhibits metastasis and epithelial-mesenchymal transition of hepatocellular carcinoma by decreasing Wnt/β-catenin signaling through targeting BCL9. Artif Cells Nanomed Biotechnol. 2019;47(1):3885–3895. 10.1080/21691401.2019.1670190 31561723

[26] Wei M, Yu H, Cai C, et al. MiR-3194-3p inhibits breast cancer progression by targeting aquaporin1. Front Oncol. 2020;10:1513. 10.3389/fonc.2020.01513 PMC7438898 32903818

[27] Tang Z, Kang B, Li C, et al. GEPIA2: an enhanced web server for large-scale expression profiling and interactive analysis. Nucleic Acids Res. 2019;47:W556–W560. 10.1093/nar/gkz430 PMC6602440 31114875

[28] Li JH, Liu S, Zhou H, et al. starBase v2.0: decoding miRNA-ceRNA, miRNA-ncRNA and protein-RNA interaction networks from large-scale CLIP-Seq data. Nucleic Acids Res. 2014 Jan;42(Database issue):D92–7. 24297251 10.1093/nar/gkt1248 PMC3964941

[29] Zhang W, Chen Q, Lei C. lncRNA MIAT promotes cell invasion and migration in esophageal cancer. Exp Ther Med. 2020 May;19(5):3267–3274. 32266022 10.3892/etm.2020.8588 PMC7132222

[30] Zhao W, Geng D, Li S, et al. LncRNA HOTAIR influences cell growth, migration, invasion, and apoptosis via the miR-20a-5p/HMGA2 axis in breast cancer. Cancer Med. 2018 Mar;7(3):842–855. 29473328 10.1002/cam4.1353 PMC5852357

[31] Livak KJ, Schmittgen TD. Analysis of relative gene expression data using real-time quantitative PCR and the 2(-Delta Delta C(T)) Method. Methods. 2001 Dec;25(4):402–408. 11846609 10.1006/meth.2001.1262

[32] Kurien BT, Scofield RH. Western blotting: an introduction. Methods Mol Biol. 2015;1312:17–30. 26043986 10.1007/978-1-4939-2694-7_5 PMC7304528

[33] Chen L, Li Q, Wang J, et al. MiR-29b-3p promotes chondrocyte apoptosis and facilitates the occurrence and development of osteoarthritis by targeting PGRN. J Cell Mol Med. 2017 Dec;21(12):3347–3359. 28609022 10.1111/jcmm.13237 PMC5706578

[34] Li Z, Qin X, Bian W, et al. Exosomal lncRNA ZFAS1 regulates esophageal squamous cell carcinoma cell proliferation, invasion, migration and apoptosis via microRNA-124/STAT3 axis. J Exp Clin Cancer Res. 2019 Nov 27;38(1):477. 31775815 10.1186/s13046-019-1473-8 PMC6882153

[35] Liang Y, Chen X, Wu Y, et al. LncRNA CASC9 promotes esophageal squamous cell carcinoma metastasis through upregulating LAMC2 expression by interacting with the CREB-binding protein. Cell Death Differ. 2018 Nov;25(11):1980–1995. 29511340 10.1038/s41418-018-0084-9 PMC6219493

[36] Cappiello F, Casciaro B, Mangoni ML. A novel in vitro wound healing assay to evaluate cell migration. J Vis Exp. 2018 Mar;17(133):56825. 10.3791/56825 PMC5931780 29608162

[37] Chen J, Yu Y, Li H, et al. Long non-coding RNA PVT1 promotes tumor progression by regulating the miR-143/HK2 axis in gallbladder cancer. Mol Cancer. 2019 Mar 2;18(1):33. 30825877 10.1186/s12943-019-0947-9 PMC6397746

[38] Xu M, Chen X, Lin K, et al. The long noncoding RNA SNHG1 regulates colorectal cancer cell growth through interactions with EZH2 and miR-154-5p. Mol Cancer. 2018 Sep 28;17(1):141. 30266084 10.1186/s12943-018-0894-x PMC6162892

[39] Jiang Y, Cao W, Wu K, et al. LncRNA LINC00460 promotes EMT in head and neck squamous cell carcinoma by facilitating peroxiredoxin-1 into the nucleus. J Exp Clin Cancer Res. 2019 Aug 20;38(1):365. 31429766 10.1186/s13046-019-1364-z PMC6700841

[40] Yao Q, Yang J, Liu T, et al. Long noncoding RNA MALAT1 promotes the stemness of esophageal squamous cell carcinoma by enhancing YAP transcriptional activity. FEBS Open Bio. 2019;9(8):1392–1402. 10.1002/2211-5463.12676 PMC6668371 31116509

[41] Chen YS, Xu YP, Liu WH, et al. Long noncoding RNA KCNMB2-AS1 promotes SMAD5 by targeting miR-3194-3p to induce bladder cancer progression. Front Oncol. 2021;11:649778. 34026626 10.3389/fonc.2021.649778 PMC8138055

[42] Ferlay J, Soerjomataram I, Dikshit R, et al. Cancer incidence and mortality worldwide: sources, methods and major patterns in GLOBOCAN 2012. Int J Cancer. 2015 Mar 1;136(5):E359–86. 25220842 10.1002/ijc.29210

[43] Cech TR, Steitz JA. The noncoding RNA revolution-trashing old rules to forge new ones. Cell. 2014 Mar 27;157(1):77–94. 24679528 10.1016/j.cell.2014.03.008

[44] Xu LJ, Yu XJ, Wei B, et al. LncRNA SNHG7 promotes the proliferation of esophageal cancer cells and inhibits its apoptosis. Eur Rev Med Pharmacol Sci. 2018 May;22(9):2653–2661. 29771415 10.26355/eurrev_201805_14961

[45] Wu C, Wang Z, Tian X, et al. Long non-coding RNA DDX11-AS1 promotes esophageal carcinoma cell proliferation and migration through regulating the miR-514b-3p/RBX1 axis. Bioengineered. 2021;12(1):3772–3786. 10.1080/21655979.2021.1940617 PMC8806645 34281459

[46] Yan S, Xu J, Liu B, et al. Long non-coding RNA BCAR4 aggravated proliferation and migration in esophageal squamous cell carcinoma by negatively regulating p53/p21 signaling pathway. Bioengineered. 2021;12(1):682–696. 10.1080/21655979.2021.1887645 PMC8291806 33602031

[47] Li W, Zhang L, Guo B, et al. Exosomal FMR1-AS1 facilitates maintaining cancer stem-like cell dynamic equilibrium via TLR7/NFκB/c-Myc signaling in female esophageal carcinoma. Mol Cancer. 2019 Feb 8;18(1):22. 30736860 10.1186/s12943-019-0949-7 PMC6367809

[48] Wu D, He X, Wang W, et al. Long noncoding RNA SNHG12 induces proliferation, migration, epithelial-mesenchymal transition, and stemness of esophageal squamous cell carcinoma cells via post-transcriptional regulation of BMI1 and CTNNB1. Mol Oncol. 2020 Sep;14(9):2332–2351. 32239639 10.1002/1878-0261.12683 PMC7463312

[49] Xu J, Ma J, Guan B, et al. LncRNA HCP5 promotes malignant cell behaviors in esophageal squamous cell carcinoma via the PI3K/AKT/mTOR signaling. Cell Cycle. 2021 Jul;20(14):1374–1388. 34190001 10.1080/15384101.2021.1944512 PMC8344760

[50] Ma L, Zhang L, Guo A, et al. Overexpression of FER1L4 promotes the apoptosis and suppresses epithelial-mesenchymal transition and stemness markers via activating PI3K/AKT signaling pathway in osteosarcoma cells. Pathol Res Pract. 2019 Jun;215(6):152412. 31000382 10.1016/j.prp.2019.04.004

[51] Huang Y, Xiang B, Liu Y, et al. LncRNA CDKN2B-AS1 promotes tumor growth and metastasis of human hepatocellular carcinoma by targeting let-7c-5p/NAP1L1 axis. Cancer Lett. 2018 Nov 28;437:56–66. 30165194 10.1016/j.canlet.2018.08.024

[52] Cheng D, Deng J, Zhang B, et al. LncRNA HOTAIR epigenetically suppresses miR-122 expression in hepatocellular carcinoma via DNA methylation. EBioMedicine. 2018 Oct;36:159–170. 30195653 10.1016/j.ebiom.2018.08.055 PMC6197532

[53] Qi X, Zhang DH, Wu N, et al. ceRNA in cancer: possible functions and clinical implications. J Med Genet. 2015 Oct;52(10):710–718. 26358722 10.1136/jmedgenet-2015-103334

[54] Winter S, Buffa F, Silva P, et al. Relation of a hypoxia metagene derived from head and neck cancer to prognosis of multiple cancers. Cancer Res. 2007;67(7):3441–3449. 10.1158/0008-5472.CAN-06-3322 17409455

[55] Favaro E, Bensaad K, Chong M, et al. Glucose utilization via glycogen phosphorylase sustains proliferation and prevents premature senescence in cancer cells. Cell Metab. 2012;16(6):751–764. 10.1016/j.cmet.2012.10.017 23177934

